# The efficiency of natural wound healing and bacterial biofilm inhibition of *Aloe vera* and Sodium Chloride toothpaste preparation

**DOI:** 10.1186/s12906-022-03548-7

**Published:** 2022-03-12

**Authors:** La-ongthong Vajrabhaya, Suwanna Korsuwannawong, Nisarat Ruangsawasdi, Chareerut Phruksaniyom, Ratchaporn Srichan

**Affiliations:** 1 Endodontics Section, College of Dental Medicine, Rangsit University, Pathumthani, 12000 Thailand; 2 Research Office, Faculty of Dentistry, Mahidol University, No.6, Yothi road, Bangkok, 10400 Ratchathewi District Thailand; 3 Department of Pharmacology, Faculty of Dentistry, Mahidol University, No.6, Yothi road, Bangkok, 10400 Ratchathewi District Thailand

**Keywords:** *A. vera*, Bacterial biofilm inhibition, Sodium chloride, Toothpaste, Wound healing

## Abstract

**Background:**

Prevention is a preliminary focus of periodontitis treatment. Rather than giving complicated treatment to a periodontitis patient, a variety of toothpastes have been suggested to prevent periodontal disease progression. Herbal toothpastes containing natural plant components for maintaining or increasing healing might be a treatment modality for improving oral hygiene. *Aloe vera* is a medicinal plant with active ingredients that have antioxidant and anti-inflammatory effects. Additionally, increased sodium in the environment inhibits microorganism growth. A toothpaste containing salt and aloe vera may be an option to provide good oral hygiene.

**Aim:**

To assess the in vitro cell migration of human gingival fibroblasts and antimicrobial effects of an herbal toothpaste containing *A. vera* and Sodium chloride.

**Methods:**

The cytotoxicity of 0.02% or 0.2% toothpaste solution on human gingival fibroblast cell line was evaluated using a 3-(4,5-dimethylthiazol-2-yl)-2,5-diphenyl-2H-tetrazolium bromide (MTT) assay. The cell migration after treatment with 0.2% (v/v) toothpaste was determined using a Boyden Chamber assay. The effect of the toothpaste on inhibiting *Porphylomonas gingivalis* planktonic and biofilm growth was compared with Chlohexidine (CHX) using a Disk Diffusion and Biofilm susceptibility test, respectively. The results of the cytotoxicity assay, inhibition zone and percentage of live cells in the biofilm were statistically analyzed with One-way analysis of variance. Cell migration and biofilm inhibition were evaluated using the independent sample t-test and multiple t-test, respectively (*p* = 0.05).

**Results:**

Neither test concentration of the toothpaste solution was toxic to the target cells. The 0.2% concentration was selected for the cell migration experiment. The herbal toothpaste formulation significantly increased cell migration compared with the control group (culture medium) (*p* = *.02*) The antimicrobial effect of this formulation on the *P. gingivalis* planktonic form was lower compared with 0.12% CHX (positive control group), however, it demonstrated greater *P. gingivalis* biofilm formation inhibition compared with the 0.12% CHX group.

**Conclusions:**

The alternative use of an herbal toothpaste instead of a non-herbal toothpaste formulation should be considered for promoting oral health care. However, further clinical studies are necessary before it can be considered for patient use.

## Background

Bacteria are the major cause of infection of the tooth structure and periodontal tissues in the oral cavity [1]. Dental caries and periodontal diseases affect a patient’s oral health. Reducing the pathogenic microbial load is a main focus of oral health treatment. To achieve this goal, toothpastes containing antimicrobial agents, such as Tricolsan, Fluoride, or Chlorhexidine, have been developed and evaluated [2]. Furthermore, the use of herbal products to eliminate oral microorganism-based diseases and aid in developing preventing drug resistance has gained interest. Various commercial chemotherapeutic agents have been incorporated in home use products for oral health care, however, phytotherapeutic agents are another option in preventing and treating oral diseases. Awareness of the minimal toxicity and less harmful effects of herbal or plant-derived medicine has generated increased investigation into using traditional herbal toothpastes rather than conventional or non-herbal toothpastes containing chemical antimicrobial agents [3].

Periodontal disease results from the host immune response to the bacteria and bacterial products in the biofilm/plaque that forms on the teeth [4]. A toothpaste containing herbal ingredients that effectively inhibits biofilm/plaque formation would reduce the incidence and severity of dental caries and periodontal diseases. *Porphylomonas gingivalis (P. gingivalis*) a Gram-negative anaerobic bacteria, is the major pathogen in periodontitis. This bacterium is one of the species involved in plaque formation. *P gingivalis* is a late colonizer of the subgingival biofilm and is associated with several destructive periodontal diseases, including periodontitis and peri-implantitis [5]. The pathogenicity of periodontal diseases includes tissue colonization by bacteria, destruction, and a defective host defense system [6]. A natural substance from a plant with antibiofilm activity against *P. gingivalis* could be included in a toothpaste. Furthermore, an herbal toothpaste with antibiofilm and wound healing promoting effects could increase patients’ oral health.

*Aloe vera* (*A. vera*) is a medicinal plant that is commonly used to treat acute or chronic wounds. It is nontoxic and can maintain and increase fibroblast migration [7]. *A. vera* is a succulent plant and is used in folk medicine for treating burns, diabetes, and digestive problems [8]. Moreover, the polysaccharides in *A. vera* gel reduce the bacterial load by stimulating phagocytic leucocytes to destroy the bacteria [9]. A toothpaste containing *A. vera* demonstrated antimicrobial potential on oral microorganisms, such as *Streptococcus mutans* and *Candida albicans* [10]. Moreover, a significant reduction in plaque accumulation from a mouth rinse containing *A. vera* was also revealed [11]. Plaque index score improvement was also reported after using a toothpaste containing *A. vera* comparable to those including tricosan in the composition [3].

Considering that antibacterial and promoting cell migration effects are beneficial in treating dental disease, the present study was designed to investigate in vitro whether a herbal toothpaste containing *A.vera* and sodium chloride had both properties, which would be important for its clinical use in managing periodontal diseases during and after periodontal treatment.

The objective of this study was to evaluate the effect of an herbal toothpaste formulation on *P.gingivalis* planktonic and biofilm growth and in vitro human gingival fibroblast cell migration.

## Materials and methods

### Cell migration

The herbal toothpaste used in this study was Herbal Salt. (Twin Lotus Co., Ltd, Bangkok, Thailand). The active ingredients in this toothpaste are *Aloe vera*, *Clinacanthus nutans,* Orange Jessamine, Hydrocotyl, Toothbrush Tree, Mangosteen, and Sodium Chloride.

### Test materials

#### Toothpaste preparation

200 mg toothpaste was mixed with 1 ml Dulbecco's Modified Eagle Medium (DMEM) (Gibco, BRL, Gaithersburg, MD, USA). The mixture was blended in a centrifuge tube at 8000 rpm for 2 h and passed through a 0.22 µm syringe filter to achieve a 20 gm% (w/v) solution.

#### Control test material preparation

Polyvinyl chloride sheets (PVC, Hatano Research Institute, Food and Drug Safety Center, Kanagawa, Japan) (3 cm^2^/2 ml media) were used as a positive control as recommended by ISO 10993–5 [12]. The sheets were sterilized by soaking in 70% alcohol for 1 min, washed in normal saline solution for 1 min, and left to dry. The films were immersed in DMEM and incubated at 37 °C in a 5% CO_2_ atmosphere for 24 h before testing. Thermanox plastics cover slips (NUNCTM, Naperville, IL, USA) were used as a negative control. The Thermanox plastics cover slips were cut into 6 cm^2^ pieces, soaked in 2 ml DMEM, and incubated in a 5% CO_2_ atmosphere at 37 °C for 24 h before testing.

### Cell culture

Human gingival fibroblast cells (ATCC® CRL-2014TM) were used in this study. The cells were maintained in DMEM supplemented with 10% fetal calf serum and antibiotics (200 µl/ml-1 penicillin G, 200 µg/ml-1 streptomycin, and 2 µg/ml-1 fungizone) and cultured at 37 °C and maintained at 95% relative humidity and 5% CO_2_. The medium was changed every other day. When the cells reached confluence, they were detached using 0.2% (w/v) trypsin and transferred to new culture flasks at a ratio of 1:4. The cells were subcultured until there were sufficient cells for the experiments. Cells from the 5–10^th^ passages were used for the experiments.

### Toothpaste cytotoxicity assay

The cytotoxicity assay was performed according to ISO 7405 [13]. The cells were seeded in 96-well culture plates at a concentration of 1 × 10^4^ cells/well. Each well contained a 100 µl cell suspension, and the plates were incubated for 24 h at 37 °C in a 5% CO_2_ atmosphere to obtain a monolayer culture. After 24 h, the media was removed from each well. Then, 100 µl of the 0.02% or 0.2% toothpaste solution were added. The positive control or negative control was placed into 96-well culture plate wells (8 wells/test material). After incubating for 24 h at 37 °C in a 5% CO_2_ atmosphere, the viability of the cells in each well was assessed using the 3-(4,5-dimethylthiazol-2-yl)-2,5-diphenyl-2H-tetrazolium bromide (MTT) assay.

The experiments were performed in triplicate. The non-toxic concentration of the toothpaste was selected for the cell migration experiment in the Boyden chamber assay.

### Boyden chamber assay

The cells were trypsinized and plated in the top of the transwell inserts in a 24-well transwell culture plate at a concentration of 2.5 × 10^5^ cells/well/200 µl serum-free medium. The lower chamber of the transwell culture plate contained 750 µl culture medium with 5% fetal bovine serum with 0.2% (v/v) toothpaste. Toothpaste was not added in the media in the control group wells and the plates were incubated at 37 °C in a 5% CO_2_ atmosphere. After 16 h, the media were removed from each well. The cells were fixed with 100% methanol at room temperature for 10 min and stained with 10% Giemsa stain for 1 h at room temperature. After 1 h, the Giemsa stain was removed by rinsing with distilled water. The non-migrating cells on the top of the transwell were removed by wiping with cotton. Images of the migrating cells on the bottom of the transwell were taken at 12 random positions using a microscope and the migrated cells were counted using Image J version 2.0. The experiment was performed using 3 wells/group and repeated five times.

### Antibacterial effect study

#### Toothpaste slurry preparation

The toothpaste slurries were obtained by mixing the toothpaste with sterile distilled water at a 1:3 (w/w) ratio as previously described [14] to simulate the concentrations of the toothpaste that would be applied in the oral cavity [15]. After centrifugation at 5,000 g for 10 min at 10 °C (Beckman J2-MC Centrifuge, California, United States) to remove any particulate matter, each supernatant was collected and passed through a 0.2 µM polyethersulfone membrane filter (Millipore, Massachusetts, United States). The filtered slurries were used as the primary solution for all tests.

#### *P. gingivalis* culture

The bacterium used in this study was *P gingivalis* (ATCC33277), which was grown in anaerobe basal agar (Oxoid, Hampshire, Unites Kingdom) with 5% sheep blood (BD Diagnostic Systems, Germany) at 37 °C for 72 h in an anaerobic jar (Oxoid) before preparing the inoculum in each experiment.

#### Disk diffusion test

The disk diffusion method was used to determine the antibacterial effect of the toothpaste formulation on *P. gingivalis*. 1–5 × 10^5^ CFU/ml of bacteria in anaerobe basal broth (Oxoid) were spread on anaerobe basal agar with 5% whole blood formed in 9-mm glass petri dishes using a swab streaking in a back-and-forth direction to ensure an even distribution of the inoculumn. Twenty ul toothpaste slurry was dropped on the 6-mm disk before placing it on the cultured agar and then incubated at 37 °C for 72 h in an anaerobic jar. 0.12% chlorhexidine was used as a positive control. The diameter of the inhibition zone was recorded for statistical comparison.

#### Minimal inhibitory concentration (MIC)

The broth dilution assay was used to evaluate the toothpaste concentration that inhibited *P. gingivalis* growth. The toothpaste slurries were diluted 40-fold with sterile deionized water before loading 100 µl diluted solution in 96-well polystyrene flat-bottom plate wells and underwent a two-fold serial dilution until 2048-fold (Corning, New York, United States). Each well was loaded with 100 µl 1–5 × 10^7^ CFU/ml *P. gingivalis* in anaerobe basal broth before incubating at 37 °C for 72 h in an anaerobic jar. The positive control was wells with only bacteria and the negative control was wells without bacteria. Photographs were taken, and the dilution factor was recorded.

#### Biofilm formation inhibition

The microtiter plate assay was performed as described by Coffey and Anderson [16] with some modifications. Stock solutions of the toothpaste slurries and 0.12% chlorhexidine were diluted 40-fold using sterile deionized water. Two-fold serial dilutions of all solutions were prepared in 96-well polystyrene flat-bottom plates to obtain 100 µl in each well. Thus, the final concentration was diluted from 40–2048-fold of the initial concentration. The inoculum was prepared from *P. gingivalis* (1–5 × 10^7^ CFU/ml) diluted in Todd Hewitt broth (BD, Franklin Lakes, New Jersey, United States) mixed with 2.2% anaerobe basal broth, and 100 µl of the inoculum were subsequently loaded into the well. The negative and positive control were wells without bacteria and wells with only bacteria, respectively. The plate was kept at 37 °C for 72 h in an anaerobic jar. To quantify biofilm formation, the 96-well plate was inverted and gently shaken to remove free-floating bacteria. The wells were rinsed with tap water 5 times. The remaining biofilms that adhered to the walls and the bottoms of the wells were stained with 200 µl 0.1% crystal violet solution (Panreac, Barcelona, Spain), and the biofilms were incubated for 30 min at room temperature. After rinsing and drying, a 30% acetic acid solution was pipetted into each well, and the plate was shaken on an open-air platform shaker (MaxQ 2000®, Thermo Fischer Scientific) at 150 RPM for 10 min. The stained biofilm was quantified by measuring the optical density at 550 nm using a microplate reader (EPOCH, Biotek, United States). The percent biofilm formation is presented as percent inhibition, which was converted using the following equation.

$$\mathrm{\% inhibition}=\frac{{\mathrm{OD}}_{\mathrm{control}}-{\mathrm{OD}}_{\mathrm{test}}}{{\mathrm{OD}}_{\mathrm{control}}} \times 100$$

#### Biofilm susceptibility test

An MTT assay was used to determine the biofilm susceptibility. Biofilms were grown in 96-well flat-bottom plates as described above. Briefly, each well was filled with 100 µl *P. gingivalis* (1–5 × 10^7^ CFU/ml) diluted in Todd Hewitt broth mixed with 2.2% anaerobe basal broth at 37 °C for 72 h to form the biofilm. To determine the biofilm susceptibility, the culture medium was removed from each well, and the biofilms were gently washed three times with 100 µl anaerobe basal broth and 100 µL of the toothpaste slurries was loaded into each well. The same volume of 2.5% NaOCl solution and 0.12% chlorhexidine was used as positive controls. The treatments were performed with 2 min exposure, and the biofilms were washed twice using 100 µL anaerobe basal broth. The amount of live bacteria was determined using 100 µl 0.5 mg/ml 3-(4,5-dimethylthiazol-2-yl)-2,5-diphenyltetrazolium bromide (Sigma-Aldrich, Missouri, United States) in anaerobe basal broth. The biofilms were incubated at 37 °C in anaerobe condition for 2 h. Images were taken using a light microscope before dissolving the formazan crystals in the bacterial cells with dimethyl sulfoxide (Thermo Fisher scientific). The absorbance was measured using a microplate reader at 570 and 690 nm.

### Statistical analysis

The results were analyzed to determine if they had a normal distribution prior to statistical comparison. The results of the herbal toothpaste cytotoxicity assay were analyzed using SPSS 18.0 software for Windows (SPSS Inc, Chicago, Illinois, USA) using One-way analysis of variance and the results of the Boyden chamber assay was analyzed using the independent sample t-test.

The mean inhibition zone and percentage live cells in the biofilms were statistically compared using One-way Analysis of Variance with SPSS version 18.0 followed by Turkey’s test for multiple comparison. Biofilm inhibition was analyzed with the multiple t-test. Statistical significance was considered when *p*-value was less than 0.05.

## Results

### Cytotoxicity assay

**Fig. 1:**
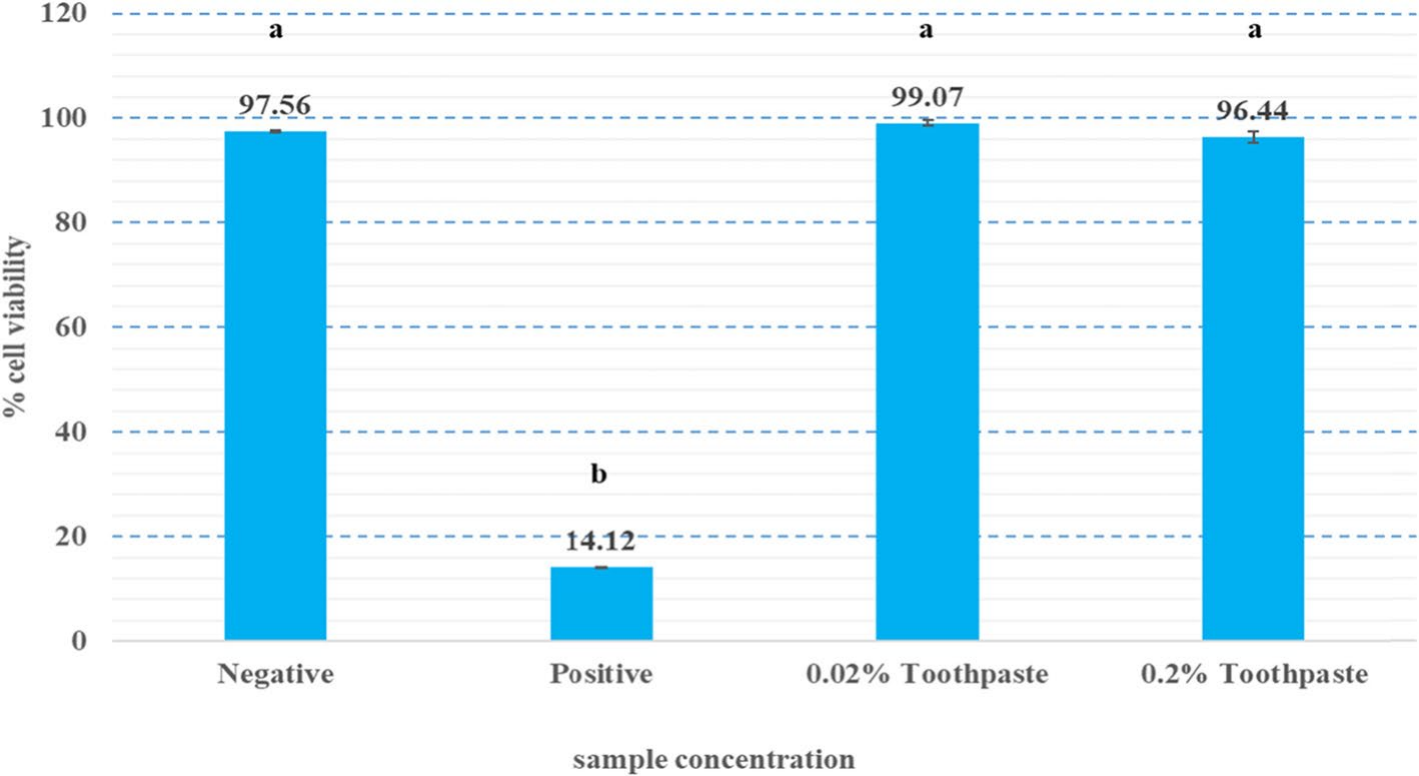
Percent cell viability in the cytotoxicity assay. HGF cell viability after exposure to the negative control, positive control, 0.02, or 0.2% Toothpaste (The same letter indicates *P >0.05*)

The effect of the herbal toothpaste on HGF cell viability is presented as percent cell viability (Fig. 1). The percentage cell viability in the 0.02% and 0.2% (v/v) toothpastes groups was 99.07% and 96.44%, respectively. Both concentrations were non-cytotoxic. Thus, 0.2% toothpaste was used in the cell migration experiments.

### Cell migration assay

**Fig. 2:**
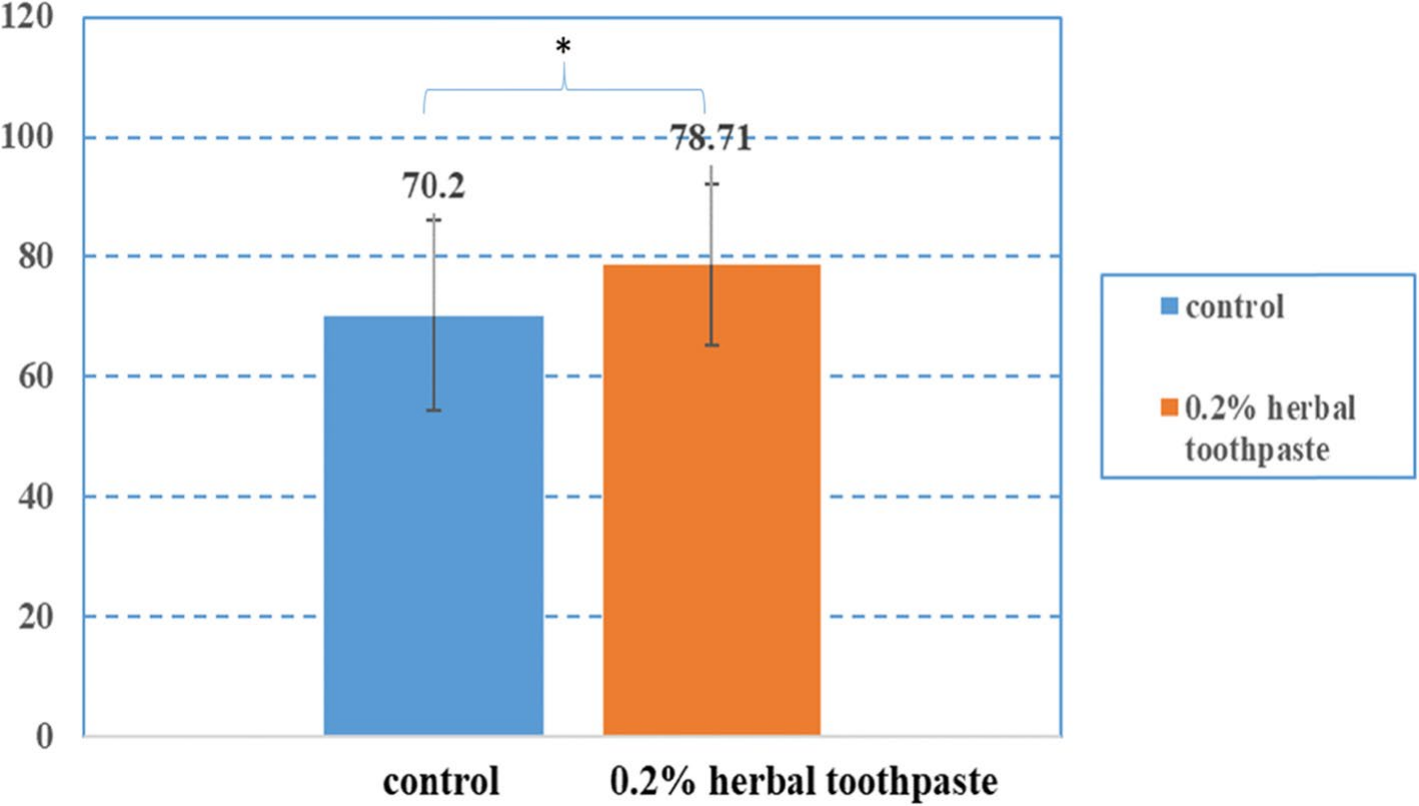
Number of migrated cells in the Boyden chamber assay. Cell migration (mean ± sd) of the herbal toothpaste through the transwell membrane in the Boyden chamber assay and control group (media only). *Indicates a significant difference between the groups (*p < 0.05*)

**Fig. 3:**
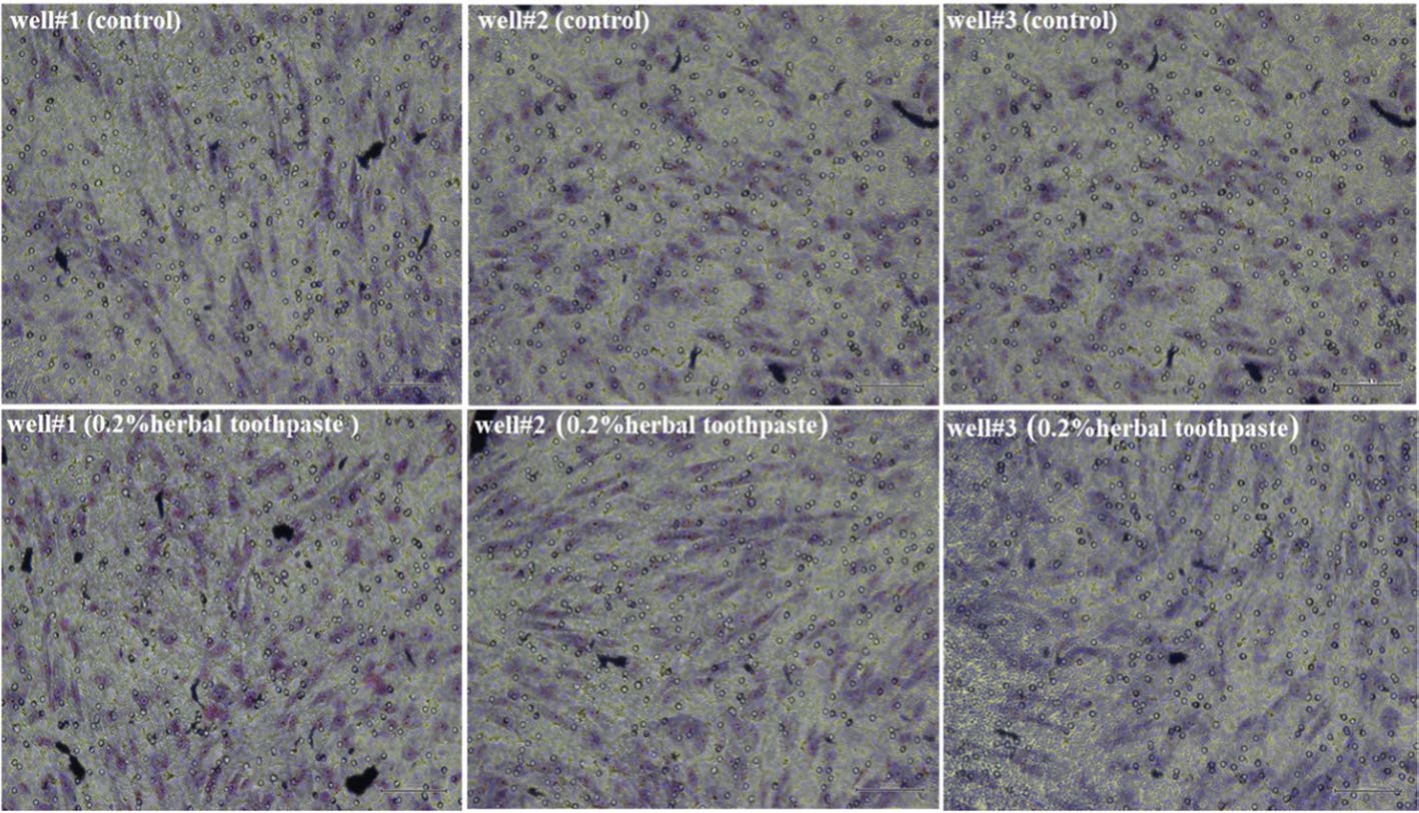
Representative images of the cell migration in the control and 0.2% herbal toothpaste groups, 3 wells/test group 10X magnification

The results of the Boyden Chamber assay indicated that the number of migrated cells after treatment with 0.2% toothpaste was 78.71 ± 13.45 cells, and the negative control was 70.20 ± 15.81 cells, which were significantly different (Fig. 2). Representative images of the cell migration assay are shown in Fig. 3.

### Antibacterial effect

#### Disk diffusion test

**Fig. 4:**
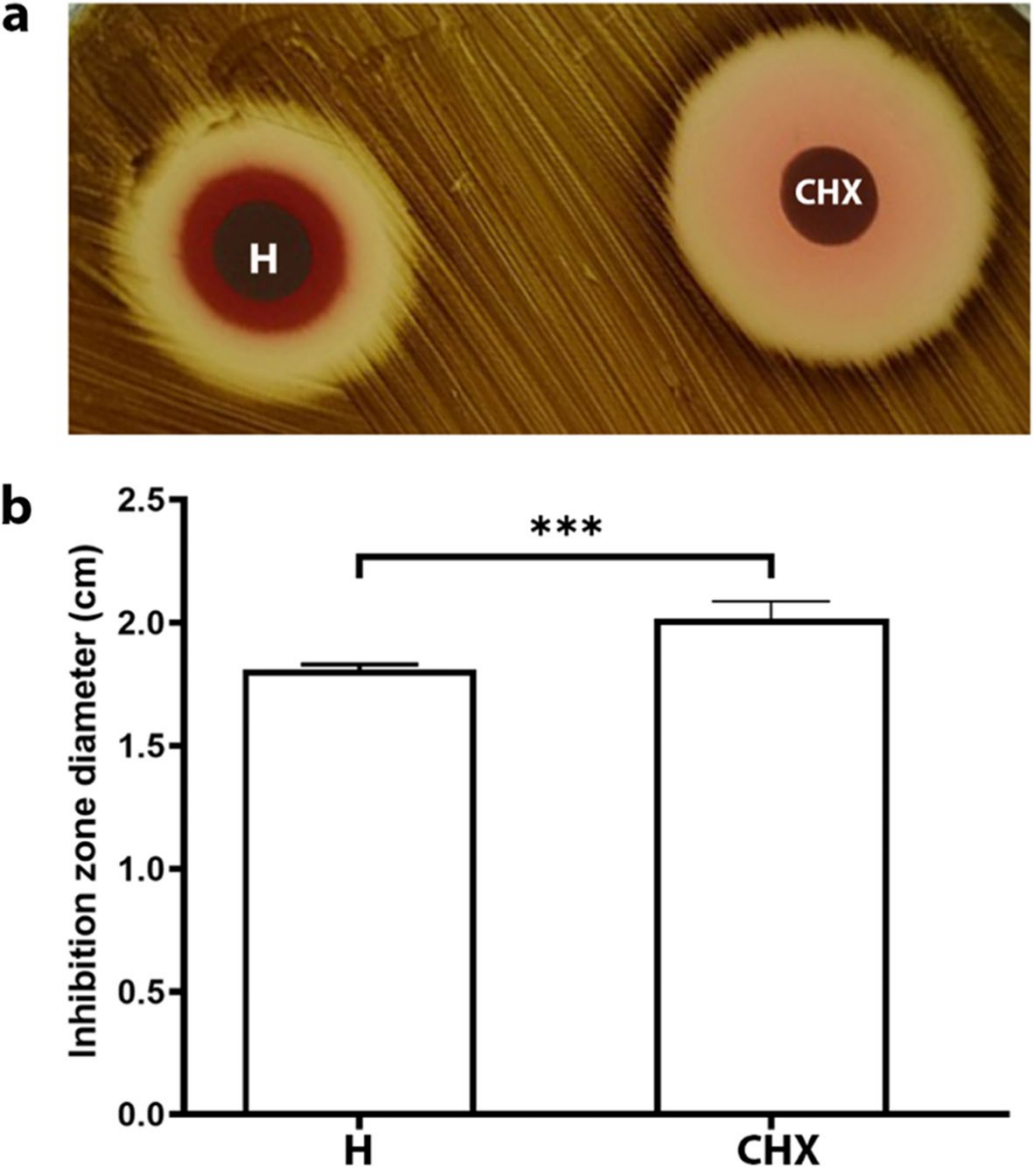
Disk diffusion test of the herbal toothpaste (H) compared with Chlorhexidine (CHX) against *P.gingivalis*. **a**) 0.12% CHX demonstrated a larger inhibition zone diameter. **b**) Statistical analysis indicated a significant difference between the groups’ inhibition zone (*p* < 0.001). *** = statistical significance compared with CHX

The zone of inhibition results (Fig. 4a) demonstrated that the 0.12% chlorhexidine group had a mean diameter of 2.02 ± 0.05 cm, which was significantly greater compared with the herbal toothpaste group (1.82 ± 0.02 cm) (Fig. 4b).

####  *Minimal inhibitory concentration*

**Fig. 5:**
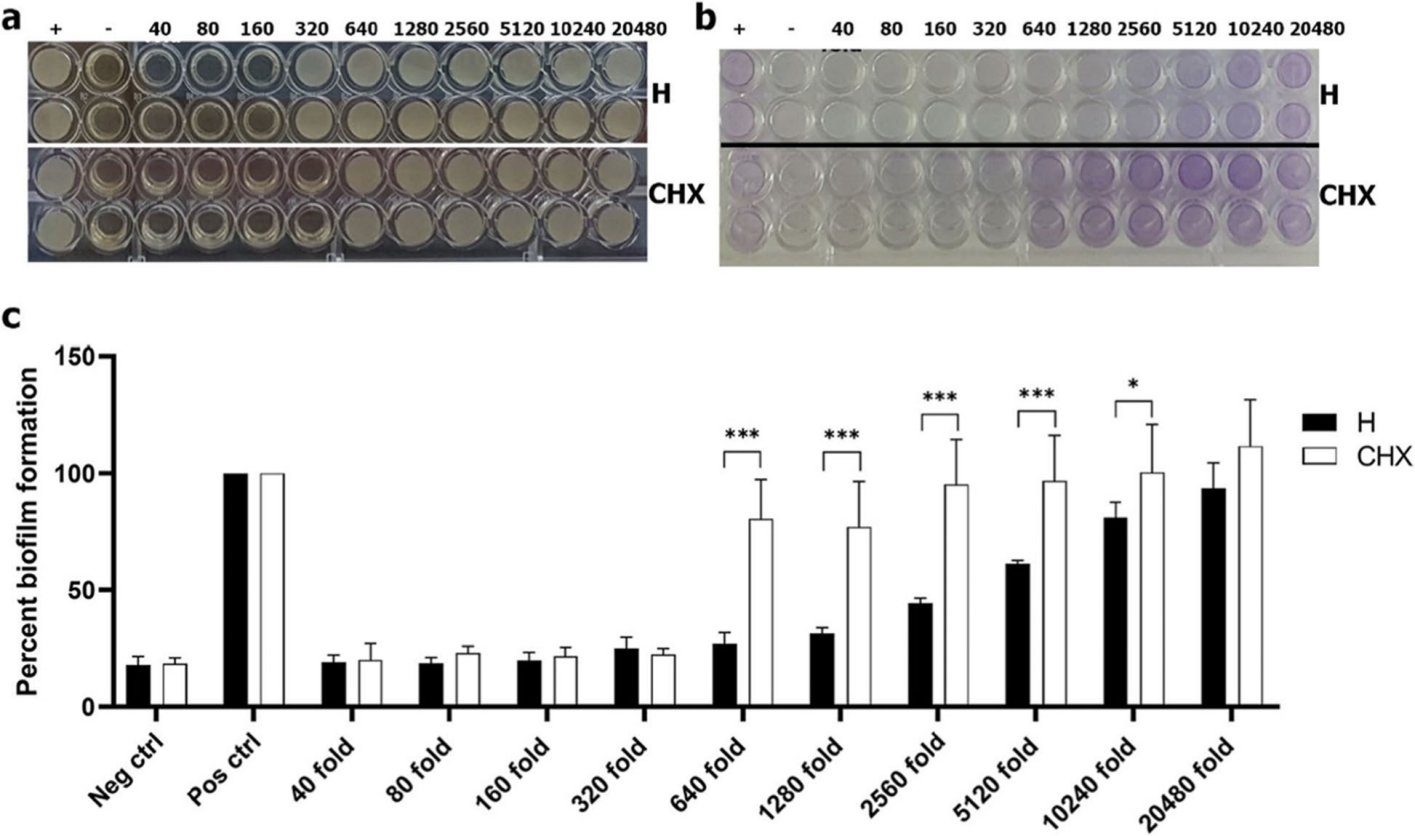
Minimal inhibitory concentration and biofilm inhibition of the herbal toothpaste against *P.gingivalis.* The toothpaste slurries and 0.12% chlorhexidine were prepared as serial dilutions from 40x–2048 × and the inhibitory effects were observed. **a**) The MIC of the H toothpaste was at the 160-fold dilution. 0.12% chlorhexidine demonstrated the lowest MIC at 320-fold dilution. **b**) A similar trend also seen in the biofilm inhbition assay. **c**) Statistical analysis indicated that at a 10,240-fold dilution H toothpaste slurry exhibited a significant higher biofilm inhibition compared with 0.12% chlorhexidine. *** = *p* < 0.001, * = *p* < 0.05. H = Herbal toothpaste

The herbal toothpaste slurries were less effective in inhibiting planktonic *P. gingivalis* growth compared with 0.12% chlorhexidine, which had an MIC at the 320-fold dilution. In contrast, the herbal toothpaste slurries demonstrated inhibited *P. gingivalis* growth at the 160-fold dilution (Fig. 5a).

#### Biofilm formation inhibition

The tested toothpaste slurries at a 40-fold dilution inhibited *P. gingivalis* biofilm formation. However, the minimal concentration that impeded biofilm formation was different between the herbal toothpaste and chlorhexidine. The biofilm amount of the herbal toothpaste gradually increased from the 640–20,480-fold toothpaste dilution (Fig. 5b, c), and the herbal toothpaste demonstrated a significantly lower amount of biofilm compared with the 0.12% CHX from 640-fold dilution to the 5,120-fold dilution (*p* < 0.001). The ability of the toothpaste to inhibit biofilm was significantly different from 0.12% chlorhexide through the 10,240-fold dilution (*p* < 0.05). Moreover, the inhibitory effect of 0.12% chlorhexidine on biofilm formation dilution-dependently declined from the 320–20,480-fold dilution.

#### Biofilm susceptibility test

**Fig. 6:**
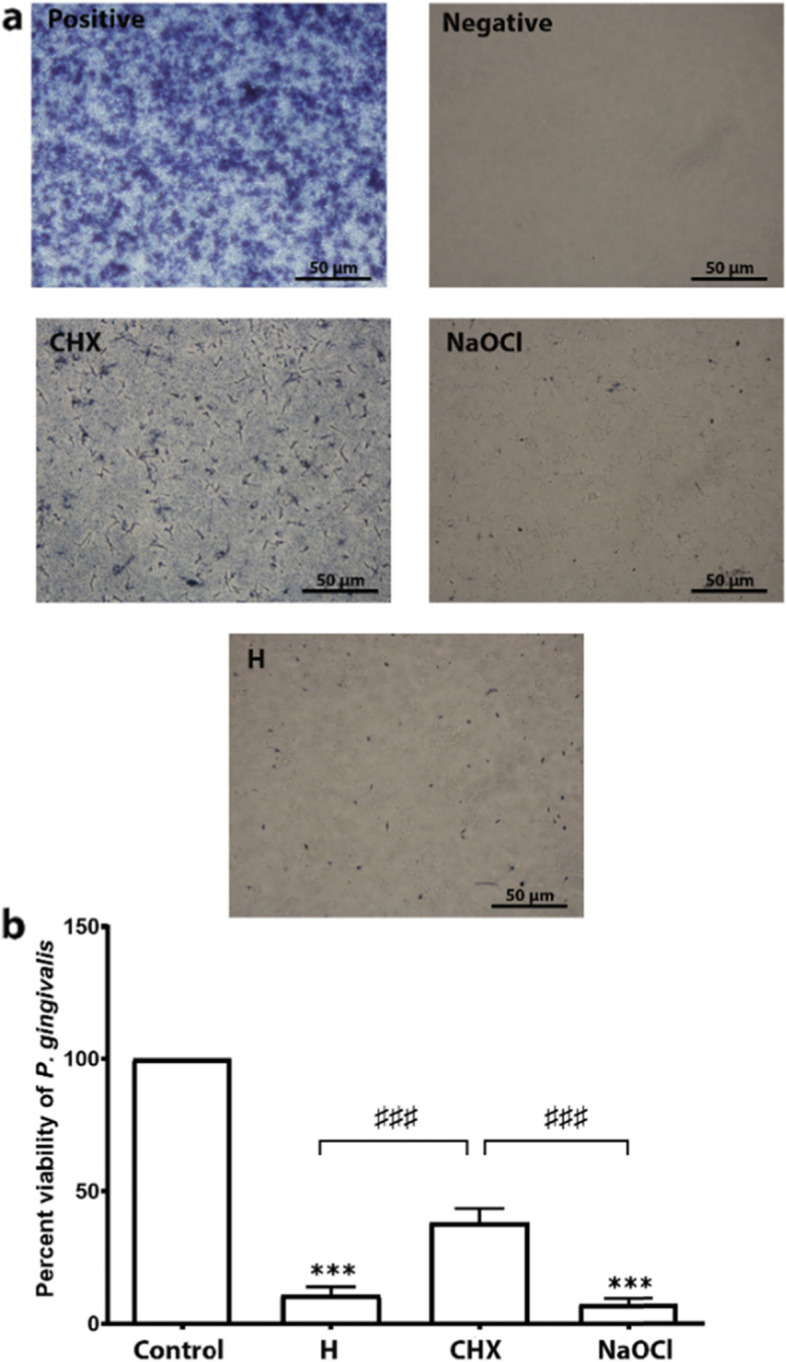
Susceptibility of the herbal (H) toothpaste against *P.gingivalis* compared with CHX, NaOCl and the control. **a**) The H toothpastes slurries, 0.12% chlorhexidine, and 2.5% sodium hypochlorite killed *P.gingivalis* in the biofilms. The control was the non-treated biofilm. Representative images of the blue formazan crystals revealed high viability of the bacteria in the control and chlorhexidine groups. **b**) Statistical comparison between the groups indicated that the H toothpaste had a higher efficacy to kill *P. gingivalis* than CHX, but less than sodium hypochlorite *** = *p* < 0.001 compared with control, ^♯♯♯^ = *p* < 0.001 compared with CHX toothpaste

We determined efficacy of the herbal toothpaste, 2.5% sodium hypochlorite, and 0.12% chlorhexidine to kill *P. gingivalis* in the biofilm using the MTT assay. The no treatment group *P. gingivalis* viability result was set at 100% viability. The results demonstrated that 7.42 ± 5.52% of *P. gingivalis* was alive after the 2.5% sodium hypochlorite treatment, which was the greatest reduction among the treatment groups. Statistical analysis revealed that the biofilm treated with toothpaste results (10.82 ± 5.52%) were similar to those of the 2.5% sodium hypochlorite group. In contrast, both of these groups exhibited significantly fewer live bacteria compared with the 0.12% chlorhexidine (38.22 + 9.13%) group (Fig. 6).

## Discussion

The present study evaluated the effect of an herbal toothpaste formulation on in vitro human gingival fibroblast cell migration/wound healing and *P.gingivalis* planktonic and biofilm growth. We found that the herbal toothpaste containing *A.vera* and Sodium Chloride increased cell migration and inhibited *P. gingivalis* biofilm formation.

The scratch assay is the original assay used for assessing cell migration. The Boyden chamber assay is a newer migration/chemotactic assay that eliminates some of the disadvantages of the scratch assay. However, a study indicated that there is no significant difference between the two assays when evaluating cell migration [17]. The Boyden chamber is a commercially available chamber with a transwell insert that is placed in the well of a cellture plate. Cells were seeded on top of the transwell and 0.2% (v/v) of toothpaste was placed in the lower chamber. The chemoattractant agent induces cells to migrate through the porus membrane to the bottom of the insert.

Cell migration evaluated using the Boyden Chamber assay demonstrated that 78.71 ± 13.45 cells in the herbal toothpaste group migrated from the upper insert through the membrane compared with 70.20 ± 15.81 cells in the control group (DMEM media *p* = *0.02*) *A. vera* is widely used in food, healthcare and medicinal industries. A previous study demonstrated that tropical *A.vera* gel stimulated wound healing by accerelating cell proliferation in a diabetic mouse model [8]. Most studies have focused on the antibacterial effect of *A. vera* on plaque and gingivitis control [18, 19]. Our investigation is the first study showing the promotion of wound healing in vitro by an herbal toothpaste with *A.vera* in the composition. Tooothpaste containing *A. vera* may have a role in maintaining or promoting gingival health during or after oral treatment. However, a future clinical study will be required to confirm this hypothesis.

Toothpaste has been incorporated with various chemical or herbal agents to enhance its antimicrobial activity. In this study, the disk diffusion test was a simple standard method used to screen the antimicrobial effect of the herbal toothpaste compared with 0.12% chlorhexidine, which is widely accepted to be a standard regimen for inhibiting *P.gingivalis* growth [20, 21]. The inhibition zone diamter when using 0.2% chlorhexidine has been shown to vary from 23.7–18.7 mm [20, 21]. Our results using 0.12% chlorhexidine demonstrated an inhibition zone with an average size of 20.2 mm, which is in the range observed in these studies. Many commercial toothpastes have demonstrated inhibitory effects against *P.gingivalis,* which is a common pathogen for oral diseases [22, 23]. Similarly, the herbal toothpaste formulae demonstrated an inhibition zone. However, the efficiency was lower than 0.12% chlorhexidine. This might be due to using a 1:3 diluted toothpaste slurry. Previous studies agree with our results that sodium chloride did not promote *P.gingivalis* growth, [24] while aloe vera gel has demonstrated inhibitory effects against *P.gingivalis* [18].

The broth dilution assay is also a common and simple method used to determine the lowest concentration or MIC of an antimicrobial agent in bacterial growth inhibition. The herbal toothpaste in our study impeded growth in the planktonic bacterial suspension. The herbal toothpaste slurries had a lower inhibitory effect against *P.gingivalis* compared with 0.12% chlorhexidine. These results correspond with those of the disk diffusion test. However, the broth dilution assay only evaluates the effect on planktonic bacteria, whose properties and conditions are dissimilar to the complex microbiota of the biofilms. The destruction of single bacterial cells in solution is easy and does not need penetration of the antimicrobial agent to kill bacteria residing in the biofilm. Further study on biofilm inhibition should be performed to elucidate the antibacterial efficiency of the herbal toothpaste.

*P.gingivalis* biofilm formation is a multistep process. Live bacterial cells adhere on the culture surface using fimbriae before developing into a complex biofilm containing nutrients and microorganism. The biofilm inhibition test allows for evaluating the efficiency of the herbal toothpaste against *P.gingivalis* adhesion. Our results indicated that the herbal toothpaste was more efficient in inhibiting *P.gingivalis* biofilm formation compared with 0.12% chlorhexidine, which is the antiseptic of choice broadly recommended for dental use. However, chlorhexidine is reported to kill *P.gingivalis* in the planktonic form better than in the biofilm, and its mechanism is to eradicate the bacteria before the biofilm is established [25].

*P.gingivalis* in the biofilm form is more virulent because bacterial genetic mutation occurs up to 18% after biofilm formation resulting in therapeutic resistance [26]. Various antibiotics and antiseptics are inadequate to kill *P.gingivalis* in the biolfilm. Chlorhexidine partly destroys bacteria in the biofilm, and the remainig bacteria can survive using carbohydrates and proteins to expand the biofilm mass [27]. Therefore, sodium hypochlorite was also used in our biofilm susceptibility test as a standard agent that has been well documented to kill bacterial residing in the biofilm [28]. However, sodium hypochlorite is highly toxic to oral mucosal cells and gingival cells, which is why there is no daily oral care product containing sodium hypochlorite. Our results were in agreement that sodium hypochlorite demonstrated greater antibacterial efficiency than chlorhexidine. The herbal toothpaste slurry showed a high ability to kill *P.gingivalis* in the biofilm, which was similar to sodium hypochlorite.

These results indicate that the use of the herbal toothpaste might have equal or superior efficiency compared with chlorhexidine in preventing and treating periodontal disease. A toothpaste with that has both an antibacterial effect and promotes wound healing promotion is an alternative choice for oral care that can provide oral health benefits for periodontal tissue and caries prevention.

A limitation of the present study is that it was performed in vitro using only an individual bacteria strain, which is unlike the actual complex biofilm in the oral cavity. Moreover, the wound healing assay evaluated the cell migration of a cell monolayer and did not involve the immune response in the oral cavity. Additional studies should be performed to confirm the clinical effectiveness of the herbal toothpaste.

## Conclusion

This in vitro study revealed that the herbal toothpaste containing *A.vera* increased cell migration and inhibited *P. gingivalis* biofilm formation. An in vivo study is necessary before this toothpaste can be widely used in patients.

## Data Availability

All data generated or analyzed during this study are included in this article.

## Abbreviations

*A.vera*: *Aloe vera*
*P. gingivalis*: *Porphyromanas gingivalis*
HGF: Human gingival fibroblast cell
CHX: Chlorhexidine
PVC: Polyvinyl chloride
ISO: International Organization for Standardization
rpm: Revolution per minute
ATCC®: The American Type Culture Collection
NaOCl: Sodium hypochlorite
